# The C-terminus of IGFBP-5 suppresses tumor growth by inhibiting angiogenesis

**DOI:** 10.1038/srep39334

**Published:** 2016-12-23

**Authors:** Jae Ryoung Hwang, Young-Jae Cho, Yoonna Lee, Youngmee Park, Hee Dong Han, Hyung Jun Ahn, Je-Ho Lee, Jeong-Won Lee

**Affiliations:** 1 Samsung Biomedical Research Institute, Samsung Medical Center, Sungkyunkwan University School of Medicine, Seoul 06351, Korea; 2 Department of Obstetrics and Gynecology, Samsung Medical Center, Sungkyunkwan University School of Medicine, Seoul 06351, Korea; 3 Institute for Refractory Cancer Research, Samsung Medical Center, Seoul, Korea; 4 Department of Immunology, School of Medicine, Konkuk University, Chungju 27478, Korea; 5 Center for Theragnosis, Biomedical Research Institute, Korea Institute of Science and Technology, Seongbuk-Gu, Seoul 02792, Korea; 6 Cancer Center, Cha Bundang Hospital, Seongnam-si, Gyeonggi-do 13496, Korea; 7 Department of Health Sciences and Technology, SAIHST, Sungkyunkwan University, Seoul, Korea

## Abstract

Insulin-like growth factor-binding protein 5 (IGFBP-5) plays a role in cell growth, differentiation, and apoptosis. In this study, we found that *IGFBP5* was markedly downregulated in ovarian cancer tissue. We investigated the functional significance of IGFBP-5 as a tumor suppressor. To determine functional regions of IGFBP-5, truncation mutants were prepared and were studied the effect on tumor growth. Expression of C-terminal region of IGFBP-5 significantly decreased tumor growth in an ovarian cancer xenograft. A peptide derived from the C-terminus of IGFBP-5 (BP5-C) was synthesized to evaluate the minimal amino acid motif that retained anti-tumorigenic activity and its effect on angiogenesis was studied. BP5-C peptide decreased the expression of VEGF-A and MMP-9, phosphorylation of Akt and ERK, and NF-kB activity, and inhibited angiogenesis in *in vitro* and *ex vivo* systems. Furthermore, BP5-C peptide significantly decreased tumor weight and angiogenesis in both ovarian cancer orthotopic xenograft and patient-derived xenograft mice. These results suggest that the C-terminus of IGFBP-5 exerts anti-cancer activity by inhibiting angiogenesis via regulation of the Akt/ERK and NF-kB–VEGF/MMP-9 signaling pathway, and might be considered as a novel angiogenesis inhibitor for the treatment of ovarian cancer.

Ovarian cancer is one of the leading causes of cancer-related death among malignancies in women because it is typically diagnosed at an advanced stage, resulting in a high death rate, high recurrence rate, and low 5-year survival^1^,^2^. The poor prognosis, clinical heterogeneity, and lack of available therapeutic strategies create major challenges for the treatment of this disease. Identification of novel therapeutic targets and the development of agents that target them are urgently needed for the treatment of refractory ovarian cancer.

Since angiogenesis is an important mechanism of tumor growth and metastasis, various angiogenesis inhibitors are broadly used to treat cancer patients. Inhibitors of the angiogenic factor, vascular endothelial growth factor (VEGF), are actively under development as cancer therapeutics^3^,^4^. In particular, bevacizumab, an antibody that binds to VEGF, is widely used for cancer therapy and is being used in combination with conventional chemotherapeutic agents for the treatment of patients with ovarian cancer^5^. However, the survival benefit of bevacizumab combined with anti-cancer drugs in patients with ovarian cancer is limited and novel anti-angiogenesis agents are therefore required.

Insulin-like growth factor binding protein 5 (IGFBP-5) is an IGFBP family member that interacts with IGF-I and IGF-II. IGFBP-5 was initially demonstrated to inhibit IGF function in cell growth, survival, and differentiation by blocking the interaction between IGF and the IGF receptor^6^; however, an IGF-independent function of IGFBP-5 has subsequently been reported^7^. The expression level of IGFBP-5 in solid tumors differs depending on cancer type^5^,^8^,^9^,^10^,^11^ and in some cancers the expression level of IGFBP-5 has been suggested as a prognostic marker^12^,^13^,^14^.

IGFBP-5 is composed of three domains: the amino (N)-terminal, carboxyl (C)-terminal, and L (linker) domains^6^. IGF-binding sites are present in the N- and C-terminal domains. The C-terminal domain also has a nuclear localization signal and a heparin-binding site. The L-domain also contains a heparin-binding site, but this site has been reported to be nonfunctional^15^. The heparin-binding domain consists of the consensus amino acid sequence XBBBXXBX or XBBXBX (B: basic amino acid, X: undefined amino acid). Many proteins, including growth factors, cytokines, enzymes, protease inhibitors, and IGFBP family proteins, are known to contain heparin-binding sites^16^.

In this study, we aimed to evaluate the functional significance of IGFBP-5 as a tumor suppressor in ovarian cancer using *in vitro* and *in vivo* systems. We found that peptides containing the heparin-binding domain derived from the C-terminus of IGFBP-5 inhibited angiogenesis and tumor growth by regulation of the Akt/ERK and NF-kB–VEGF/MMP-9 signaling pathway in an IGF-independent manner. Our results suggest that a peptide derived from the C-terminus of IGFBP-5 may serve as a novel angiogenesis inhibitor for the treatment of ovarian cancer.

## Results

### IGFBP-5 expression was downregulated in ovarian cancer tissues and its overexpression decreased cell survival

**Figure 1:**
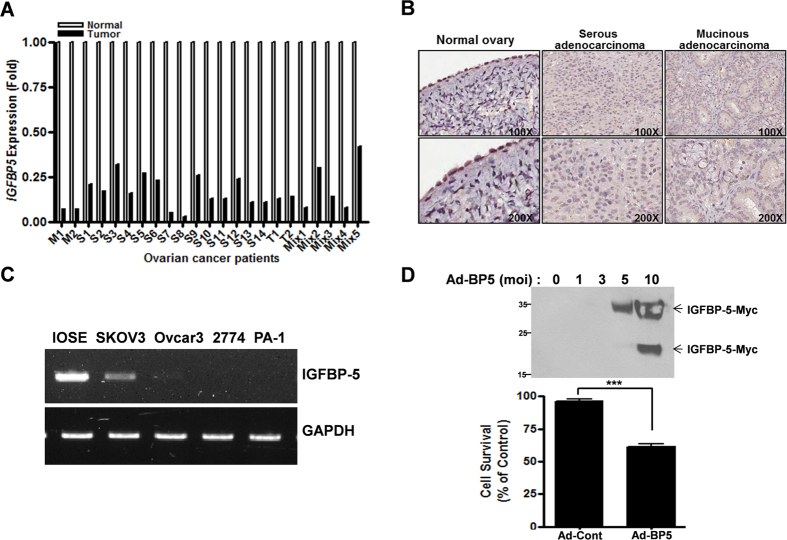
IGFBP-5 is downregulated in ovarian cancer and its overexpression inhibits cell survival. (**A**) cDNA microarray analysis of *IGFBP5* in ovarian cancer tissues. Gene expression level in ovarian carcinomas is expressed as fold-change relative to levels in normal tissues, normalized to *GAPDH* expression (*P* < 0.001). ‘M’ represents mucinous type of ovarian cancer, ‘S’ represents serous type, ‘T’ represents transitional cell, and ‘Mix’ represents mixed type ovarian carcinomas. Different numbers indicate different patients. (**B**) Representative immunohistochemical staining of IGFBP-5 in ovarian cancer and normal ovarian tissues. (**C**) *IGFBP5* expression in ovarian cancer cell lines and IOSE normal ovarian cells was determined by RT-PCR. (**D**) MTT assay was performed to determine cell death after adenoviral expression of IGFBP-5 in 2774 cells. Data represent the mean ± SEM (***P < 0.001). Expression of Myc-tagged IGFBP-5 by adenovirus was detected by western blotting using anti-Myc antibody.

We analyzed global gene expression in ovarian cancer tissues obtained from 30 ovarian cancer patients using cDNA microarray analysis and found that *IGFBP5* expression was significantly downregulated in ovarian cancers compared with normal ovarian tissues (Fig. 1A). We represented the result of cDNA microarray anaylsis from 23 ovarian cancer tissues except borderline ovarian cancers in Fig. 1A. Moreover, using immunohistochemical staining for IGFBP-5, we detected expression in the nuclei of cells in normal ovarian epithelium, but not in ovarian cancer tissue (Fig. 1B). We examined the *IGFBP5* expression level in several ovarian cancer cell lines using reverse transcription PCR (RT-PCR) and found that it was downregulated in cancer cell lines relative to levels in the IOSE normal ovarian cell line (Fig. 1C). Adenovirus expressing IGFBP-5 was prepared and used to infect the MDAH 2774 (hereafter referred to as 2774) ovarian cancer cell line, which expresses very low levels of *IGFBP5*. Adenoviral IGFBP-5 expression was confirmed by western blotting using an anti-Myc antibody (Fig. 1D, upper panel). A truncated Myc-IGFBP-5 was also detected by western blotting when adenovirus was infected with multiplicity of infection (MOI) = 10. The viability of 2774 cells infected with adenoviruses expressing either vector or *IGFBP5* (MOI = 5) was measured in 72 h after infection by MTT assay, which revealed that infection with adenovirus expressing *IGFBP5* resulted in increased cell death relative to infection with adenovirus expressing vector only (Fig. 1D, lower panel).

### Ectopic expression of the C-terminal region of IGFBP-5 inhibits tumor growth

**Figure 2:**
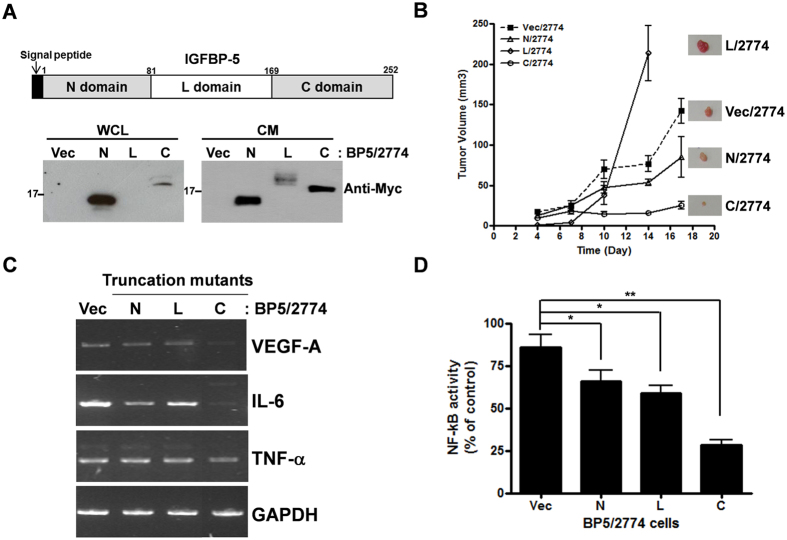
Ectopic expression of IGFBP-5 C-terminal domain inhibited tumor growth and VEGF gene expression. (**A**) Schematic diagram of each domain of IGFBP-5 and expression analysis of each truncation mutant of IGFBP-5 in the 2774 stable cell lines. WCL indicates whole cell lysate and CM indicates conditioned media. (**B**) 2774 cell lines stably expressing each truncation mutant were subcutaneously injected into nude mice (n = 4 per group) and tumor growth was measured. Tumors obtained from each mouse are shown on the graph. The experiment was repeated three times with similar results. (**C**) RT-PCR analysis of *VEGF-A* expression was performed in each 2774 stable cell line. RT-PCR was repeated at least three times with similar results. (**D**) Each 2774 stable cell line expressing a truncation mutant was transfected with plasmids expressing NF-kB-RE-luciferase and renilla luciferase to measure NF-kB activity. The assay was performed three times in duplicate and data represent the mean ± SEM (*P < 0.05, **P < 0.01).

As each domain of IGFBP-5 performs different functions, we investigated which domain is responsible for the inhibition of tumor growth. We prepared 2774 stable cell lines expressing a single domain of IGFBP-5 (N, L, or C-domain). Expression of each truncation mutant was confirmed by western blotting using whole cell lysates and conditioned media (Fig. 2A). To determine the functional region of IGFBP-5 responsible for the inhibition of tumor growth, each 2774 stable cell line was subcutaneously injected into nude mice. Tumor growth was attenuated in mice injected with 2774 cells stably expressing the C-terminal domain (C/2774) compared with mice injected with cells expressing vector or the N- or L-domain (Fig. 2B). To study the mechanism underlying this effect of the C-terminal domain, we analyzed expression of the cytokines, VEGF-A, interleukin 6 (IL-6), and tumor necrosis factor alpha (TNF-α), key genes for tumor growth, by RT-PCR. Intriguingly, gene expression of VEGF-A, IL-6, and TNF-α was downregulated in C/2774 cells (Fig. 2C). Since these genes are involved in the nuclear factor-kB (NF-kB) signaling pathway, which is a critical pathway in tumor growth^17^,^18^, we measured NF-kB activity in the 2774 stable cell lines and showed that it was inhibited by 60–70% in C/2774 cells relative to its activity in Vec/2774 cells (Fig. 2D).

### A peptide derived from the C-terminal region of IGFBP-5 inhibited VEGF gene expression and NF-kB activity

**Figure 3:**
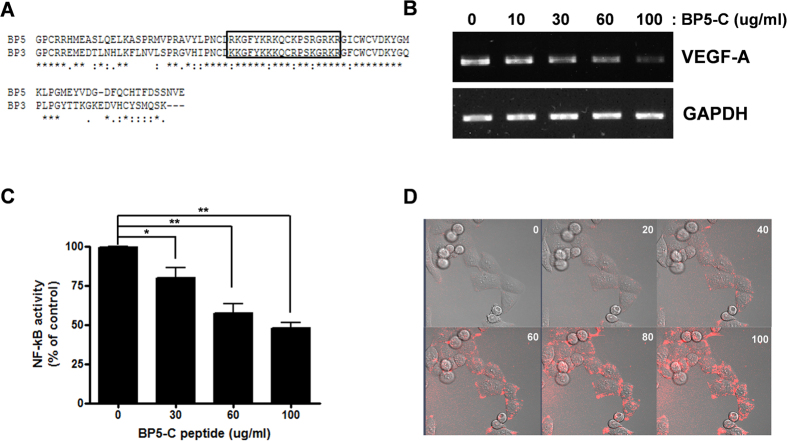
BP5-C peptide was taken up into 2774 cells and inhibited *VEGF-A* expression. (**A**) Alignment of amino acid sequences of the C-terminal domains of IGFBP-5 and IGFBP-3. The amino acid sequence of the synthetic peptide is shown in the box. (**B**) RT-PCR analysis of *VEGF-A* expression in 2774 cells treated with increasing amounts of BP5-C peptide. (**C**) NF-kB activity was measured by dual luciferase assay. The assay was performed three times in duplicate and data represent the mean ± SEM (*P < 0.05, **P < 0.01). (**D**) Internalization of BP5-C peptide in 2774 cells was monitored by incubation with rhodamine-labeled peptide and observed with a live-cell imaging system. Numbers indicate incubation time. The experiment was repeated three times.

To identify the specific functional region of the IGFBP-5 C-terminus, we compared the C-terminal sequence of IGFBP-5 with that of IGFBP-3, which has been reported to be an inhibitor of angiogenesis in several cancers^19^,^20^. We identified 32 amino acids in the IGFBP-5 C-terminus that were highly homologous to a region in IGFBP-3 as determined by CLUSTALW multiple alignments (Fig. 3A). Rombouts *et al*. reported that a peptide derived from 18 of these 32 amino acids could enter cells^21^. Therefore, we synthesized an 18-amino acid peptide and tested whether this peptide could inhibit *VEGF-A* expression in 2774 cells. As shown in Fig. 3B, IGFBP-5 C-terminal-derived peptide (BP5-C) inhibited *VEGF-A* expression in a dose-dependent manner. BP5-C peptide also inhibited NF-kB activity in a dose-dependent manner, as measured with an NF-kB-dependent luciferase reporter assay (Fig. 3C). To determine whether the BP5-C peptide entered 2774 ovarian cancer cells, rhodamine-labeled BP5-C peptide was incubated with 2774 cells and its entry into cells was monitored by live-cell image microscopy. As shown in Fig. 3D, rhodamine-labeled BP5-C peptide bound to cells by 40 min of incubation and entered into the cells.

### BP5-C inhibits Akt/ERK and NF-kB-VEGF signaling pathway in an IGF-independent manner

**Figure 4:**
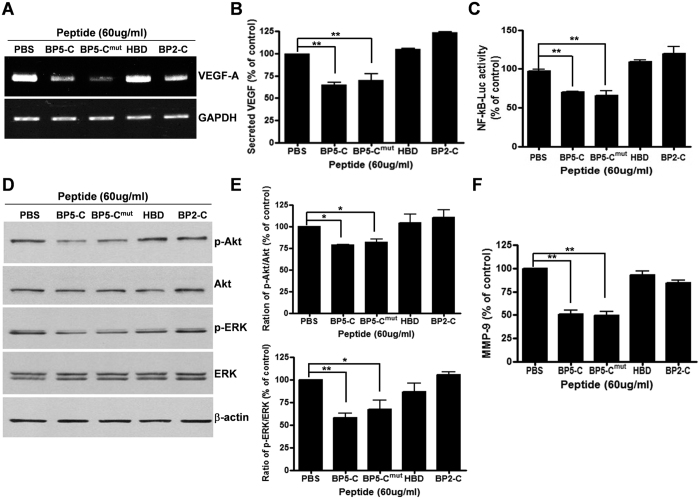
BP5-derived peptides inhibited the Akt/ERK and NF-kB–VEGF/MMP-9 signaling pathway. (**A**) 2774 cells were treated with each peptide and RT-PCR was performed to quantitate *VEGF-A* expression. (**B**) Secreted VEGF-A in the conditioned media was measured by VEGF-A-specific ELISA kit. (**C**) NF-kB activity was determined after incubation of 2774 cells with each peptide. (**D**) At 30–32 h after administration of peptides to 2774 cells, cells were lysed in RIPA buffer and phosphorylation of Akt and ERK was analyzed by western blotting using anti-phospho-specific antibody. Total Akt and ERK were determined by western blotting using anti-Akt and anti-ERK antibodies and β-actin was measured as a protein loading control. (**E**) Expression level of p-Akt and p-ERK was quantified by densitometric analysis of western blots from three different experiments and the expression ratio of p-Akt to Akt (upper panel) and p-ERK to ERK (lower panel) was represented in a bar graph. (**F**) Secreted MMP-9 in the conditioned media was measured using a MMP-9-specific ELISA kit. All experiments were performed at least three times, and representative data are presented. All data represent the mean ± SEM (*P < 0.05, **P < 0.01).

We hypothesized that downregulation of VEGF-A by the BP5-C peptide might be due to blockage of the interaction between IGF and the IGF receptor because the BP5-C peptide contains an IGF-binding site. Therefore, to determine whether the IGF-binding site of IGFBP-5 is necessary for the inhibition of VEGF-A, we prepared a mutant peptide with a mutation in the IGF-binding site (BP5-C^mut^) (see Methods). As IGFBP-2 has been studied as an oncogene and as a prognostic marker for ovarian cancer^3^,^22^, a peptide derived from the C-terminal region of IGFBP-2 containing its IGF-binding site (BP2-C) and a peptide derived from its heparin-binding domain (HBD)^23^ were also synthesized as controls. The BP5-C and BP5-C^mut^ peptides decreased *VEGF-A* expression in 2774 cells compared with levels in cells treated with the HBD and BP2-C peptides (Fig. 4A). We also measured secretion of VEGF-A by ELISA and found that the amount of secreted VEGF-A following treatment with BP5-C or BP5-C^mut^ was reduced by 25–30% compared with those following treatment with PBS or the HBD or BP2-C peptides (Fig. 4B). We also showed that the BP5-C and BP5-C^mut^ peptides inhibited NF-kB activity by 30% relative to levels in PBS-treated cells (Fig. 4C). We next studied the effect of BP5-C on the upstream factors of NF-kB signaling by measuring the amount of phosphorylated Akt/protein kinase B and MAP kinase (ERK) by western blot analysis using phospho-specific antibodies (Fig. 4D). The results of western blot analysis obtained by scanning the bands were represented in a bar graph (Fig. 4E upper panel and lower panel). Phosphorylation of Akt and ERK was decreased by 25–20% and 40–35%, respectively, in the presence of BP5-C and BP5-C^mut^ compared with PBS-treated cells. Secretion of MMP-9/gelatinase B, which increases cell migration and invasion and thereby increases angiogenesis^24^,^25^, was determined by MMP-9-specific ELISA. Incubation of 2774 cells with BP5-C and BP5-C^mut^ peptides resulted in a 50% reduction in the secretion of MMP-9 compared with PBS-treated cells (Fig. 4F). We determined whether expression of Activator protein-1 (AP-1) component (c-jun and c-fos), which regulates *VEGF* and *MMP-9* expressions and angiogenesis^26^,^27^, was affected by BP5-related peptides by western blot analysis. BP5-derived peptides did not alter the expression level of AP-1 component (Supplementary Fig. S1). These data collectively indicate that the IGFBP-5 C-terminal domain inhibits the Akt/ERK and NF-kB–VEGF/MMP-9 signaling pathway and that this inhibition is an IGF-independent phenomenon.

### BP5-C inhibits recombinant VEGF protein in the upstream of Akt/ERK signaling pathway and inhibits NF-kB activity in a VEGF-independent manner

**Figure 5:**
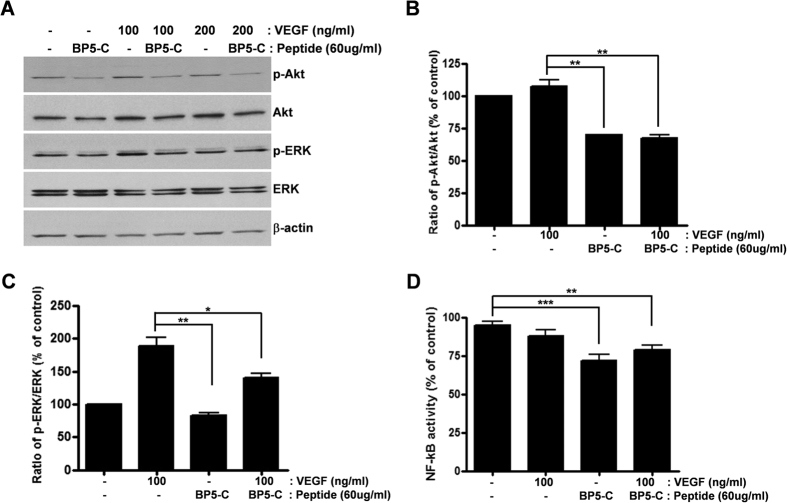
BP5-C inhibited VEGF protein in the upstream of Akt and ERK signaling pathway and inhibited NF-kB in a VEGF-independent manner. (**A**) At 30–32 h after administration of peptide and/or recombinant VEGF protein to 2774 cells as indicated, cells were lysed in RIPA buffer and phosphorylation of Akt and ERK was analyzed by western blotting using anti-phospho-specific antibody. Total Akt and ERK were determined by western blotting using anti-Akt and anti-ERK antibodies and β-actin was measured as a protein loading control. (**B**) Expression level of p-Akt was quantified by densitometric analysis of western blots from three different experiments and the expression ratio of p-Akt to Akt was represented in a bar graph. (**C**) Expression level of p-ERK was quantified by densitometric analysis of western blots from three different experiments and the expression ratio of p-ERK to ERK was represented in a bar graph. (**D**) NF-kB activity was determined after incubation of 2774 cells with VEGF alone, BP5-C peptide alone, and VEGF with BP5-C. All experiments were performed at least three times, and representative data are presented. All data represent the mean ± SEM (*P < 0.05, **P < 0.01, ***P < 0.001).

In order to determine mechanism of BP5-C inhibition on VEGF-related signaling pathway, we studied the effect of BP5-C in phosphorylation of Akt and ERK and NF-kB activity in the presence of excess amount of recombinant VEGF protein. Commonly 10–20 ng/ml of recombinant VEGF protein has been used to activate endothelial cells^28^. In our study, 100 ng/ml of recombinant human VEGF protein was used. VEGF induced phosphorylation of Akt and ERK compared with the control (Fig. 5A). BP5-C inhibited VEGF-induced phosphorylation of Akt and ERK (Fig. 5A). These results obtained by western blot analysis are shown in bar graphs (Fig. 5B and C). It was reported that treatment of VEGF protein induced NF-kB activity in endothelial cells^28^,^29^. However, although BP5-C alone inhibited NF-kB activity, VEGF could not induce NF-kB activity in 2774 cells (Fig. 5D). We tried to incubate VEGF protein for shorter time than in Fig. 5D, but induction of NF-kB activity by VEGF was not detected (data not shown). Taken together, BP5-C inhibits VEGF protein in the upstream of Akt/ERK signaling pathway, probably at extracellular level and also inhibits NF-kB activity in a VEGF-independent manner.

### BP5-C inhibits angiogenesis *in vitro* and *ex vivo*

**Figure 6:**
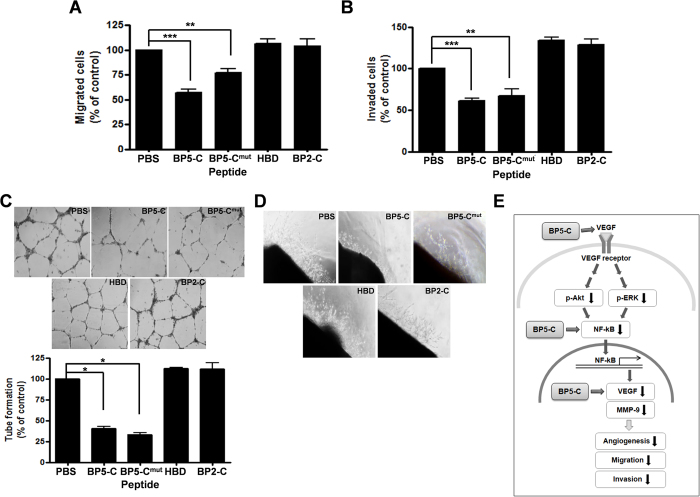
BP5-derived peptides inhibited angiogenesis *in vitro* and *ex vivo*. (**A**,**B**) Migration and invasion of HUVEC were determined after incubation with each peptide. (**C**) Tube formation of HUVEC was measured (Upper panel). Tube formation was scored by regarding three-branch points as markers of active tube formation. Lower panel represents results from three different experiments as a bar graph. (**D**) Blood vessel sprouting was measured in rat aortas after treatment with each peptide. (**E**) Schematic presentation of the angiogenesis-related signaling pathway that is inhibited by BP5-C. All assays were performed at least three times, and representative data are presented. All data represent mean ± SEM (*P < 0.05, **P < 0.01, ***P < 0.001).

Since BP5-derived peptides downregulated the Akt/ERK and NF-kB–VEGF/MMP-9 signaling pathway that is important for angiogenesis^30^, we next examined whether BP5-C has anti-angiogenic activity by examining the effect of BP5-derived peptides on human umbilical vein endothelial cell (HUVEC) migration and invasion. The BP5-derived peptides BP5-C and BP5-C^mut^ inhibited HUVEC migration by 40% and 25%, respectively, and inhibited invasion by approximately 40%, compared with PBS-treatment (Fig. 6A and B). We then assessed the effects of these peptides on HUVEC tube formation and blood vessel sprouting from rat aortas. The BP5-C and BP5-C^mut^ peptides inhibited HUVEC tube formation by 60–70% compared with PBS treatment, as scored by counting three-branch points as a marker of angiogenesis (Fig. 6C). Rat aortic blood vessel sprouting was also reduced upon treatment with BP5-C and BP5-C^mut^ compared with PBS treatment (Fig. 6D). Taken together, we proposed an angiogenesis-related signaling pathway that is inhibited by BP5-C (Fig. 6E).

### BP5-C inhibited tumor growth in a 2774 cell line xenograft mouse model

**Figure 7:**
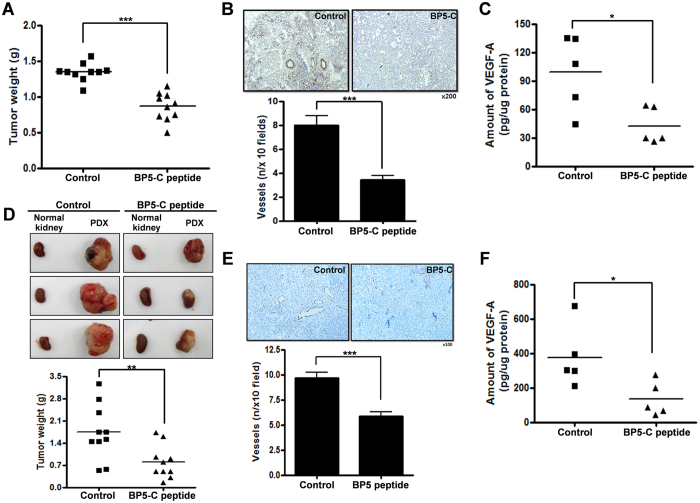
BP5-C inhibited tumor growth in ovarian cancer-xenograft mouse models. (**A**) Effect of BP5-C on tumor growth in 2774 xenograft model mice after peritoneal injection of BP5-C peptide and PBS. Tumor weight was represented as a bar graph. (**B**) Angiogenesis was quantified by counting CD31-positive vessels. (**C**) Tumors were lysed with RIPA buffer and expression of VEGF-A was measured by ELISA. All data represent the mean ± SEM (*P < 0.05, ***P < 0.001). (**D**) Effect of BP5-C on tumor growth in ovarian cancer patient-derived xenograft (PDX) mice. Upper panel presents tumor size compared with a kidney obtained from the same mouse, and lower panel shows weights of tumors obtained from BP5-C- or PBS-treated mice. (**E**) Vessel formation was measured by CD31 staining of tumors. (**F**) Expression of VEGF-A in tumor lysates obtained from PDX mice was determined using a VEGF-A-specific ELISA kit. All data represent the mean ± SEM (*P < 0.05, **P < 0.01, ***P < 0.001).

We intraperitoneally (i.p.) inoculated mice with 2774 ovarian cancer cells and then injected the mice with BP5-C peptide or PBS as a control. BP5-C peptide inhibited tumor growth by 40% compared with PBS (Fig. 7A). Immunohistochemical staining for CD31 was performed to detect blood vessels in the tumors. As shown in Fig. 7B, tumors obtained from BP5-C peptide-treated mice demonstrated an approximately 50% reduction in blood vessel formation relative to controls (upper panel). These data were presented as a bar graph representing the number of CD31-stained vessels (lower panel). Measurement of VEGF-A production in tumors by ELISA showed that VEGF-A expression was reduced by approximately 60% in BP5-C peptide-treated tumors compared with PBS-treated tumors (Fig. 7C).

### BP5-C inhibited tumor growth and angiogenesis in a patient-derived xenograft (PDX) of ovarian cancer

We developed the PDX model by subrenal implantation of human ovarian cancer tissue into BALB/C nude mice^31^. The PDX model used in this study was generated with a tumor that was histologically defined as a FIGO stage IIIC serous papillary adenocarcinoma, grade III. The patient was a 70-year-old woman who received primary debulking surgery followed by adjuvant chemotherapy with paclitaxel-carboplatin for six cycles, and no recurrence was detected until 25 months post-therapy. BP5-C peptide was i.p. injected into the mice three times per week, and the mice were sacrificed after eight injections of peptide. The tumors obtained from BP5-C peptide-injected mice were significantly smaller than those obtained from PBS-injected mice (Fig. 7D, upper panel); tumor weight was reduced by approximately 50% in BP5-C peptide-injected mice relative to PBS-injected mice (Fig. 7D, lower panel). Immunohistochemical staining for CD31 revealed the number of blood vessels in tumors obtained from BP5-C peptide-injected mice was 50% of that in controls (Fig. 7E). We also determined VEGF-A expression levels in these tumors by ELISA. As shown in Fig. 7F, VEGF-A levels were significantly decreased in tumors obtained from BP5-C peptide-injected mice compared with controls.

## Discussion

Reports on the expression and function of IGFBP-5 in various cancers have been contradictory^5^,^8^,^13^,^32^, and in this study we found that *IGFBP5* was significantly downregulated in ovarian cancer patient tissues and ovarian cancer cell lines relative to normal controls. Ectopic expression of IGFBP-5 induced cell death in ovarian cancer cells, demonstrating its function as a tumor suppressor. IGFBP-5 can be cleaved by several proteases, and as evidence of its cleavage *in vivo*, truncated forms have been found in serum^33^,^34^,^35^. Furthermore, it has been suggested that each domain of IGFBP-5 might have a different function^36^,^37^. We therefore tested the effect of the three domains of IGFBP-5 on tumor growth and demonstrated that the C-terminal domain had a tumor suppressive function and that its overexpression inhibited *VEGF-A* expression and the Akt/ERK–NF-kB signaling pathway.

We studied the signaling pathways on which BP5-C had an inhibitory effect and proposed that BP5-derived peptides containing the heparin-binding domain might be involved in the inhibition of angiogenesis in Fig. 6E. VEGF is an important cytokine in blood vessel formation and MMP-9 is a matrix metalloprotease that degrades components of the extracellular matrix thus increasing cell migration and invasion^25^,^38^. NF-kB activity is known to induce VEGF and MMP-9 expression, thereby positively regulating angiogenesis^39^,^40^. Secreted VEGF binds to its receptor and activates Akt and ERK/MAPK through downstream signaling, thereby activating NF-kB^41^,^42^. In this study, BP5-C and BP5-C^mut^ peptides reduced the expression of VEGF-A, MMP-9, and phosphorylation of Akt and ERK, and inhibited NF-kB activity. Mechanism of BP5-C inhibition in the presence of excess amount of VEGF suggests that BP5-C inhibits VEGF-related signaling pathway, at least in two points; (1) inhibits VEGF protein in upstream of Akt/ERK activation, probably in the extracellular level and (2) inhibits NF-kB activity in the cytoplasm. The exact inhibitory mechanism of BP5-C on VEGF protein needs to be further studied. Since they contain a nuclear localization signal (NLS), they may also enter the nucleus and regulate *VEGF-A* expression.

Anti-angiogenic activity of IGFBP-3 has been well studied^19^,^20^ and the sequence of BP5-C peptide is very similar with the sequence of IGFBP-3 in C-terminus. Hence, we determined whether anti-angiogenic activity of BP5-C and BP5-C^mut^ was due to the induction of IGFBP-3 gene expression in 2774 cells. We first measured *IGFBP3* expression in several ovarian cancer cell lines used in Fig. 1C and determined *IGFBP3* expression level by RT-PCR in 2774 cells treated with peptides. Although *IGFBP3* was highly expressed in IOSE normal ovarian cell line and some ovarian cancer cell lines such as Ovcar3 and SKOV3, *IGFBP3* expression was highly downregulated in 2774 and PA-1 cells (Supplementary Fig. S2A). Treatment of BP5-C and BP5-C^mut^ peptides on 2774 cells had no effect on the expression of *IGFBP3* (Supplementary Fig. S2B).

IGF is a growth factor that induces angiogenesis^43^. IGFBP-5 exerts IGF inhibitory functions by suppressing the interaction between IGF and its cognate receptor^6^. As the BP5-C peptide used in this study contains a putative IGF-1-binding site, we examined whether inhibition of VEGF-A expression and NF-kB activity by this peptide might be due to inhibition of IGF function. For this purpose, a peptide with a mutation in the IGF-binding site of BP5-C (BP5-C^mut^) and a peptide derived from the IGF-binding site of IGFBP-2 (BP2-C) were synthesized and examined in angiogenesis activity assays. BP5-C^mut^ showed the same inhibitory effect as BP5-C on the Akt/ERK and NF-kB–VEGF/MMP-9 signaling pathway and angiogenesis. Therefore, the inhibition of angiogenesis is not an IGF-dependent phenomenon. Consistent with this result, even though it contains an IGF-binding site, the C-terminal region of IGFBP-2 (BP2-C) did not inhibit angiogenesis. IGFBP-2 has been well studied with respect to its function in tumor growth and activation of angiogenesis, and is known to have a heparin-binding site^22^,^23^. To study whether other proteins with heparin-binding sites also have anti-angiogenic functions, we synthesized the HBD peptide containing the IGFBP-2 heparin-binding site. HBD peptide derived from IGFBP-2 did not inhibit angiogenesis indicating that angiogenesis inhibition is specific to the heparin-binding site of IGFBP-5. IGFBP-5 has three putative heparin-binding sites: two overlapping sites in the linker domain (^132^VKKDRRKKL^140^) and one in the C-terminal region (^205^YKRKQCKP^212^); however, only the C-terminal heparin-binding site is functional^15^. This might explain why VEGF-A expression and tumor growth were inhibited only in 2774 cells stably expressing the C-terminus of IGFBP-5. Consistent with our results, Luther *et al*. reported that the C-terminal domain of IGFBP-5 inhibited tumor growth and metastasis of human osteosarcoma^36^. It has been reported that a peptide comprising amino acids 201–218 induces mesangial cell migration^44^. However, the present study clearly demonstrated that the same peptide inhibited angiogenesis and tumor growth in two different systems of ovarian cancer-xenograft mouse: an ovarian cancer cell line xenograft mouse and an ovarian cancer patient-derived tumor-xenograft mouse. Taken together, the data indicate that the HBD of IGFBP-5 may function in a cell type-specific manner and through different mechanisms depending on cell context.

We cannot rule out the possibility that sequences in the IGFBP-5 C-terminus other than amino acids 201–218 affect the inhibition of angiogenesis and tumor suppression because expression of the entire 83 amino acids of the C-terminal domain in 2774 cells exerted a greater inhibitory effect on the expression of certain cytokines, including VEGF-A, IL-6, and TNF-α, and on tumor growth, than the synthetic peptide. This study identified a minimal amino acid motif of IGFBP-5 that retained anti-angiogenic and anti-tumorigenic activity through inhibition of Akt/ERK and NF-kB-VEGF/MMP-9 signaling pathway.

In this study, MDAH 2774 cell, an endometrioid adenocarcinoma, was used to study the effect of BP5-C on tumor growth. However, the most prevalent epithelial ovarian cancer (EOC) is the serous adenocarcinoma (60% of all EOCs)^45^. Thus, the anti-cancer activity of BP5-C on majority of EOC is uncertain. Although we performed functional study of IGFBP-5 in an *in vitro* system using only 2774 cells, our animal study using two different mice (orthopotic mice represent endometrioids and PDX mice represent serous type ovarian cancer) suggested that anti-tumorigenic activity of IGFBP-5 may be effective in EOCs overall.

In conclusion, the C-terminus of IGFBP-5 exerts a tumor suppressive function, probably by inhibiting angiogenesis. A synthetic peptide derived from the C-terminus of IGFBP-5 inhibited angiogenesis and tumor growth in an *in vitro* system and in ovarian cancer xenograft animal systems. This inhibition was IGF-independent and probably IGFBP-5 heparin-binding domain-dependent. These results suggest that a peptide derived from the C-terminus of IGFBP-5 may serve as a novel angiogenesis inhibitor for the treatment of ovarian cancer.

## Methods

### Cell culture

Ovarian carcinoma cell lines 2774, Ovcar3, SKOV3, PA-1, and HUVEC were obtained from the American Type Culture Collection (ATCC). We obtained the authentications of the cell lines from the provider and checked the certificates of the cell lines, which included test results (species verification and short tandem repeat DNA profiling assay) and procedures for authentication. The IOSE normal ovary cell line was kindly provided by Dr. Nelly Auersperg (Department of Obstetrics and Gynecology, University of British Columbia). 2774 cells were maintained in DMEM containing 10% FBS and 100 units/ml penicillin/100 μg/ml streptomycin (Invitrogen, Carlsbad, CA, USA) and were grown at 37 °C in a 5% CO_2_ incubator. HUVEC cells were maintained in EGM-2 media (EGM-2 bullet kit, Lonza, Basel, Switzerland) on gelatin-coated dishes.

### Preparation of adenovirus expressing IGFBP-5

To construct an adenoviral vector expressing Myc-tagged IGFBP-5 (Ad-BP5), IGFBP-5-Myc was amplified using two primers (F: 5′-CCGCAAGCTTATGGTGTTGCTCACCGC-3′ and R: 5′-CCGGTCTAGACCAGATCCTCTTCTGAG-3′) with IGFBP-5-Myc (in pcDNA 3.1/myc-His) as a template. The PCR product was cloned into the HindIII and XbaI sites of the pShuttleCMV vector and the resultant construct was sequenced. Adenovirus expressing IGFBP-5-Myc and the control vector were prepared by Seoulin Bioscience (Seoul, South Korea). Expression of IGFBP-5-Myc protein by Ad-BP5 after infection of 2774 ovarian cancer cells was confirmed by western blotting using anti-Myc antibody.

### Stable transfection

Truncation mutants of IGFBP-5 were prepared as described in Hwang *et al*.^37^ using pSecTag2/Hygro A vector (Invitrogen). Plasmids encoding each truncation mutant of IGFBP-5 as well as the control vector were individually transfected into 2774 cells using Lipofectamine 2000 (Invitrogen) according to the manufacturer’s instructions. Twenty-four hours after transfection, media was replaced with DMEM containing 10% FBS, 500 μg/ml G418 (Invitrogen), and 1X penicillin/streptomycin, and cells were grown until negative control cells were dead. Cells surviving in growth media containing G418 were picked and expression of the truncation mutants was confirmed by western blotting.

### Reverse transcription-PCR (RT-PCR) of cytokine gene expression and ELISA assays for VEGF and MMP-9 expression

2774 stable cell lines were washed with 1× PBS and serum starved overnight in 24-well plates. Total RNA was isolated using Trizol (Invitrogen) and used for cDNA synthesis. RT-PCR was performed using EF-Taq polymerase (Solgent Inc., Daejeon, South Korea). Primers used for IGFBP-5 and cytokine genes are as follows:

IGFBP-5 (F: 5′-GAGAAAGCCCTCTCCATGTGCCCC-3′/R: 5′-GGCCCTGCTCAGACTCCTGTCTCA-3′); VEGF-A (F: 5′-ATGAACTTTCTGCTGTCTTGGG-3′/R: 5′-CCGCCT CGGCTTGTCACA TCTG-3′); IL-6 (F: 5′-TGTAGCCGCCCCACACAGACAGCC-3′/R: 5′-GAAGAGCCCTCAGGCTGGACTGC-3′); GAPDH (F: 5′-GGAGTCCACTGGCGTCTTCACCACC-3′/R: 5′-CCTCCGACGCCTGCTTCACCACCTT-3′).

The PCR products were separated on 1% agarose gel containing SYBR safe DNA gel stain (Invitrogen, USA) and detected bands on Gel Doc XR and Image Lab software (Bio-Rad, USA).

For the VEGF and MMP-9 ELISA assays, we used overnight medium collected from cells treated peptides for 24 hours (peptides were treated for 7 hours, then changed media with freshly diluted peptide with Opti-Meme, and further incubated for overnight) and transferred to a 96-well plate for either VEGF-specific or MMP-9-specific ELISA using a Quantikine ELISA kit (R&D Systems, Minneapolis, MN, USA) according to the manufacturer’s instructions. To measure VEGF-A from tumors obtained from xenograft mice, tumors were minced under liquid nitrogen, lysed in RIPA buffer (50 mM Tris, pH 7.5, 150 mM NaCl, 1 mM ethylenediamine tetraacetic acid (EDTA), 0.25% sodium deoxycholate, 0.2% Nonidet P-40) supplemented with protease inhibitor cocktail (Roche Molecular Biochemicals, Indianapolis, IN, USA) and 1 mM phenylmethylsulfonyl fluoride (PMSF), and centrifuged for 10 min at 13,000 rpm and 4 °C. The supernatant was applied to a 96-well plate of the VEGF ELISA kit. A standard curve using recombinant VEGF protein provided in the kit was run with each assay and used to determine the concentration of VEGF.

### Immunohistochemistry

Immunohistochemical staining for IGFBP-5 was carried out on 4-μm formalin-fixed, paraffin-embedded tissue sections. After antigen retrieval in 10 mM sodium citrate buffer (pH 6.5) in a microwave followed by blocking with an Avidin/biotin blocking kit (Vector Labs, Burlingame, CA, USA), tissue slides were stained with anti-IGFBP-5 antibody (Santa Cruz Biotechnologies, Dallas, TX, USA) and biotinylated anti-mouse secondary antibody (DAKO, Denmark). IGFBP-5 was detected with avidin-conjugated HRP (DAKO) and images were scanned with a Scan Scope (Aperio, Heidelberger, Germany). To quantify angiogenesis, microvessel density was determined by counting CD31-positive vessels as described previously^46^. In brief, 8-μm thick sections of fresh-frozen tumor samples were fixed and incubated with anti-mouse CD31 antibody (Abcam, Cambridge, UK) at 4 °C overnight. CD31 staining was detected with an Envision detection system (DAKO).

### Peptide synthesis

Peptides were synthesized by Peptron Inc. (Daejeon, South Korea) without any modifications to either the N- or C-terminus and purified by HPLC. To produce rhodamine-labeled BP5-C peptide, rhodamine B was coupled to the N-terminus of the BP5-C peptide. Peptides were resuspended in 1× PBS at a concentration of 1 mg/ml, aliquoted into small volumes to prevent frequent freeze-thaw cycles, and stored at −80 °C. The peptides used in this study are as follows (amino acids in bold represent the IGF-binding site):

**BP5-C**: ^201^RK**G**FYKRK**Q**CKPSRGRKR^218^

**BP5-C**^**mut**^: ^201^RKAFYKRKACKPSRGRKR^218^

**HBD**: ^171^KHHLGLEEPKKLRPPPAR^188^

**BP2-C**: ^228^KH**G**LYNLK**Q**CKMSLNGQR^245^

### Rat aortic ring assay

Aortas were dissected from 4- to 6-week-old Sprague–Dawley rats. After removing the surrounding tissues the aortas were thoroughly rinsed with 1× PBS and cut into 1-mm ring segments. The aortic rings were immersed in Matrigel in the wells of 24-well plates. Peptides (60 μg/ml) in EGM media diluted 1:4 with EBM-2 serum-free media were added to the wells. The aortic rings were cultured at 37 °C with 5% CO_2_ and treated with each peptide every day. Growing sprouts of endothelial cells were observed and photographed using a microscope on day 3.

### Development of *in vivo* mouse xenograft models with an established cell line and patient-derived xenografts

Female BALB/c nude mice were purchased from Orient Bio, Seongnam, South Korea. This study was reviewed and approved by the Institutional Animal Care and Use Committee (IACUC) of the Samsung Biomedical Research Institute (protocol no. H-A9-003), which is accredited by the Association for the Assessment and Accreditation of Laboratory Animal Care International (AAALAC International) and abides by the guidelines of the Institute of Laboratory Animal Resources (ILAR). To establish orthotopic models, intraperitoneal injection of 2774 cells (5 × 10^6^ cells/0.2 ml Hank’s balanced salt solution) followed by intraperitoneal peptide injection after one week in BALB/C nude mice was performed. To establish the patient-derived xenograft (PDX) model of ovarian cancer, surgical patient tumor specimens were reduced to small pieces (less than 2–3 mm), implanted into the subrenal capsules of the left kidneys of BALB/C nude mice, and propagated by serial transplantation^27^. The mice used in these experiments were 6- to 8-weeks old. Peptide was intraperitoneally injected 3 months after ovarian cancer tissue transplantation. BP5-C peptide (10 mg/kg) or PBS was injected into model mice every 2–3 days for the subsequent 3 weeks. Mice (n = 10 per group) were monitored daily for tumor development by checking peritoneal cavity with hands and sacrificed when any of them appeared moribund or at the end of the treatment. The tumors were photographed and weighed, and then stained by immunohistochemistry using an anti-CD31 antibody. Tumors were fixed in formalin and embedded in paraffin, or snap-frozen in OCT compound (Sakura Finetek Japan, Tokyo, Japan) in liquid nitrogen. We recorded body weight, tumor weight, and number of tumor nodules for each mouse.

### Peptides and VEGF treatment for western blot analysis

2774 cells were plated in 6-well plates and the next day, each peptide (60 ug/ml) was treated in growth media containing 2% FBS for 7 hours, then changed media with freshly diluted peptides, and incubated for overnight. The next day, media was changed with freshly diluted peptides and incubated for 7 hours. Cells were lysed in RIPA buffer and expression of proteins was analyzed by western blotting.

For administration of VEGF protein into cells, recombinant human VEGF_165_ protein (Peprotech, USA) was used. VEGF protein was reconstituted in water to 1 mg/ml and then was diluted to 10 ug/ml concentration with 1X PBS containing 0.1% BSA as a carrier protein. VEGF with/without BP5-C peptide was treated into 2774 cells for 30–32 hours.

### NF-kB reporter assay

2774 stable cell lines were plated in 24-well plates and transfected using Lipofectamine 2000 with plasmids encoding NF-kB-responsive firefly luciferase (NF-kB-RE-Luc), and renilla luciferase (pRL-SV40), which is an internal control for normalizing transfection efficiency. Twenty-four hours after transfection, cells were washed with PBS, serum-starved in Opti-MEM overnight, and then assayed for luciferase activity using the dual-luciferase reporter assay kit (Promega, Madison, WI, USA). Peptides (each 60 ug/ml) were treated for 24 hours (7 hours-treatment and then overnight-treatment with freshly diluted peptide) before luciferase assay.

### Migration, invasion, and tube formation assays

HUVEC cells were plated in 60-mm dishes. At 24 h after plating the cells were incubated with the relevant peptides (60 μg/ml) in EGM-2 media (EGM-2 bullet kit, Lonza) for a further 48 h. Peptides were freshly administered every 24 h. For the tube formation assay, 300 μl/well of matrigel (200–300 μg/ml, BD Bioscience, San Jose, CA, USA) was added to 24-well plates and incubated for 1 h. Peptide-treated cells (1 × 10^5^ cells) in EGM media diluted with EBM serum-free media (1:4) containing 60 μg/ml peptide were added to each well containing matrigel and incubated in a 37 °C incubator for 7–16 h. Tube formation of HUVECs was observed and photographed under microscopy. Positive tube formation was scored by the presence of three-branch points. For the migration and invasion assays, before plating the cells the undersides of the Transwell inserts were coated with gelatin. For the invasion assay, the insides of the inserts were also coated with 100 μl of matrigel per insert by 1 h incubation at 37 °C in a CO_2_ incubator. Peptide-treated cells (1 × 10^4^ cells in 100 μl of serum-free EBM containing 0.5% BSA) and 60 μg/ml of each peptide were plated onto a Transwell insert and 600 μl of EGM growth media was added to the lower chamber. Cells were incubated for 48 h in a CO_2_ incubator at 37 °C. After incubation, cells were fixed with 100% methanol and stained with hematoxylin and eosin. After removal of non-invaded cells using a cotton swab the insert membrane was mounted and invaded cells were counted under a microscope (100×, Olympus BX51, Tokyo, Japan).

### Statistical analysis

The Mann–Whitney U test was used to evaluate significances when comparing differences among groups in both the *in vitro* and *in vivo* assays. All statistical tests were two-sided, and *P* values less than 0.05 were considered to be statistically significant. SPSS software (version 17.0; SPSS, Chicago, IL, USA) was used for all statistical analyses.

## Additional Information

**How to cite this article**: Hwang, J. R. *et al*. The C-terminus of IGFBP-5 suppresses tumor growth by inhibiting angiogenesis. *Sci. Rep.* **6**, 39334; doi: 10.1038/srep39334 (2016).

**Publisher's note:** Springer Nature remains neutral with regard to jurisdictional claims in published maps and institutional affiliations.

## Supplementary Material

Supplementary Information
