# Supplementary material for: The C-terminus of IGFBP-5 suppresses tumor growth by inhibiting angiogenesis
Source: Sci Rep. 2016 Dec 23;6:39334. doi: 10.1038/srep39334 (PMC5180245; doi:10.1038/srep39334)
Supplement: Supplementary Information [file srep39334-s1.pdf]

The C-terminus of IGFBP-5 suppresses tumor growth by inhibiting angiogenesis

Jae Ryoung Hwang[1,†,*], Young-Jae Cho[2,3,†], Yoonna Lee[1], Youngmee Park[1], Hee Dong Han[4], Hyung Jun Ahn[5], Je-Ho Lee[6,*] & Jeong-Won Lee[2,3,7,*]

[1]Samsung Biomedical Research Institute, Samsung Medical Center, Sungkyunkwan University School of Medicine, Seoul 06351, Korea. [2]Department of Obstetrics and Gynecology, Samsung Medical Center, Sungkyunkwan University School of Medicine, Seoul 06351, Korea. [3]Institute for Refractory Cancer Research, Samsung Medical Center, Seoul, Korea,[4]Department of Immunology, School of Medicine, Konkuk University, Chungju 27478, Korea. [5]Center for Theragnosis, Biomedical Research Institute, Korea Institute of Science and Technology, Seongbuk-Gu, Seoul 02792, Korea,[6]Cancer Center, Cha Bundang Hospital, Seongnam-si, Gyeonggi-do 13496, Korea,[7]Department of Health Sciences and Technology, SAIHST, Sungkyunkwan University, Seoul, Korea.

[†] These authors contributed equally to this work.

*Correspondence and requests for materials should be addressed to JWL (garden.lee@samsung.com), JRH (jhwang1@skku.edu), or JHL (jeholee@gmail.com)

Supplementary Information

1. Supplementary Methods

Measurement of tumor growth of 2774 cells stably transfected with truncation mutants of IGFBP-5

2774 stable cells ($1X10^6$ cells/mouse) expressing each truncation mutant of IGFBP-5 were subcutaneously injected into BALB/C nude mouse (n=4 per group) and observed tumor growth every 3-4 days by measuring the tumor volume with digital caliper (Mitutoyo, Japan). The tumor volume was calculated by $V= \pi/6 \cdot (\text{length}) \cdot (\text{width}) \cdot (\text{height})$.

Live-cell image of rhodamine-labeled BP5-C

One day before treating peptide, 2774 cells were plated onto coverglass-bottom dish (SPL, Korea). The next day, growth media containing rhodamine-labeled BP5-C (60ug/ml) was added into dish and we detected the entry of peptide into cells by a live-cell image analysis using CLSM700 (Carl-Zeiss, Germany). Cells were monitored for 2-3 h.

2. Supplementary Figures and Figure Legends

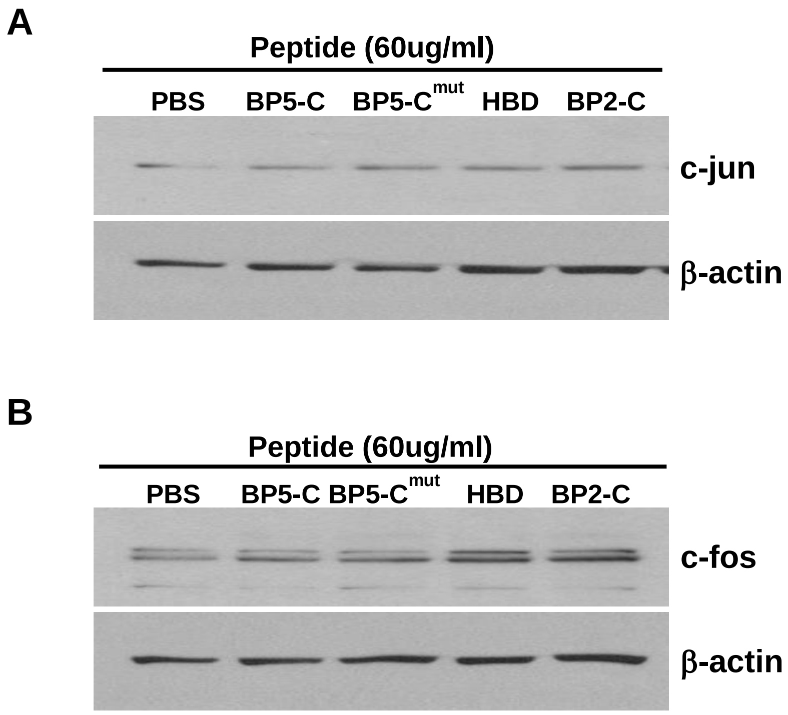

Figure S1. BP5-derived peptide had no effect on AP-1 components. (**A** and **B**) At 30-32 h after administration of peptides to 2774 cells as indicated, cells were lysed in RIPA buffer and expression of c-jun (**A**) and c-fos (**B**) was analyzed by western blotting. β-actin was measured as a protein loading control.

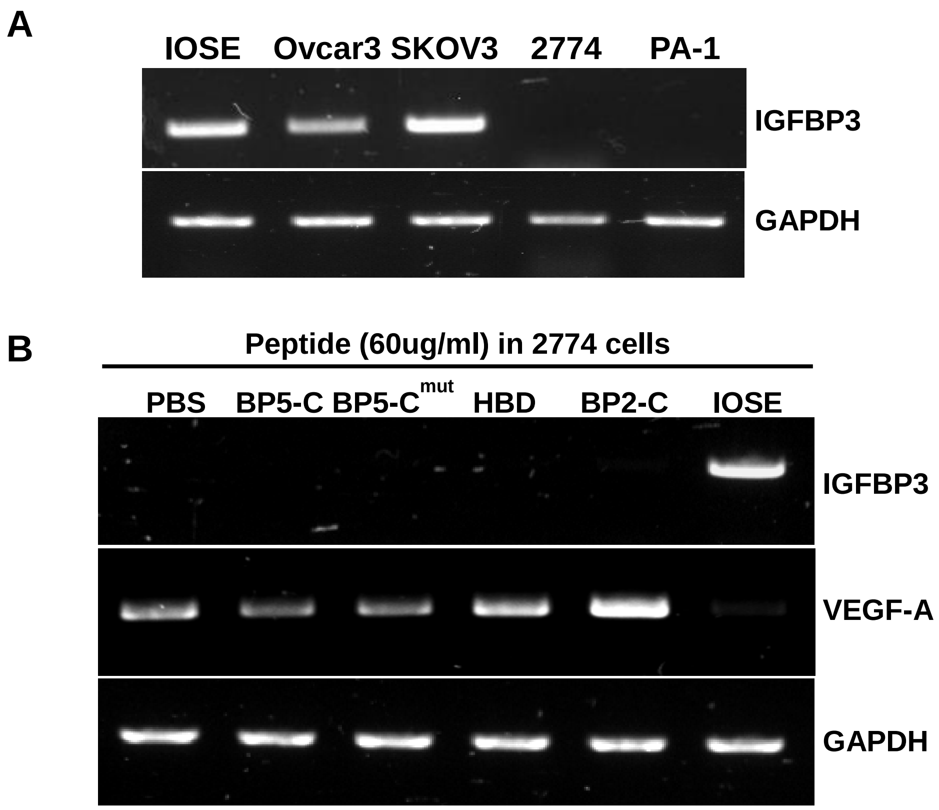

Figure S2. IGFBP-3 gene expression was not induced by BP5-derived peptides. (**A**) *IGFBP3* gene expression in ovarian cancer cell lines and IOSE normal ovarian cells was determined by RT-PCR. (**B**) BP5-derived peptides had no effect on *IGFBP3* expression in 2774 cells determined by RT-PCR. *IGFBP3* expression in IOSE cells was used as a positive control. RT-PCR for *VEGF* was used to indicate that BP5-derived peptides were active to inhibit *VEGF* expression in this condition.

3. Original western blots

1. In Figure 1D.

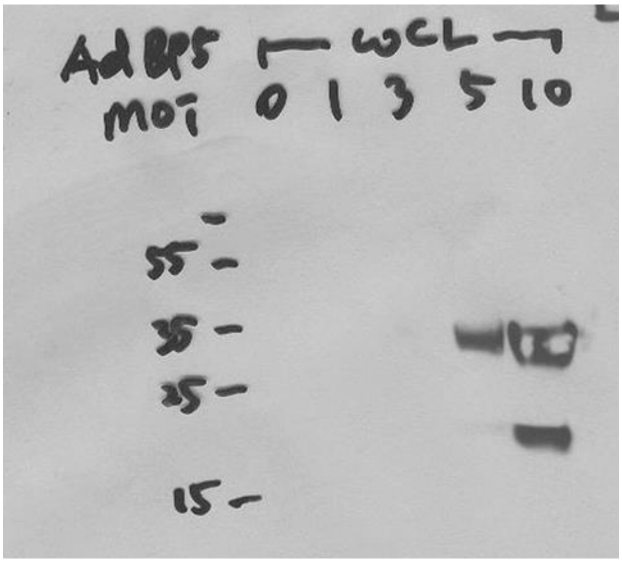

2. In Figure 2A

Left pannel

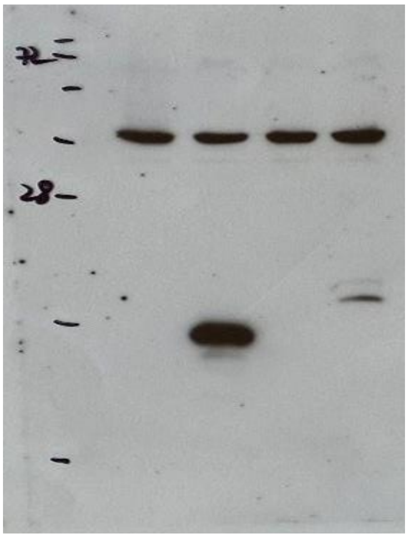

Right pannel

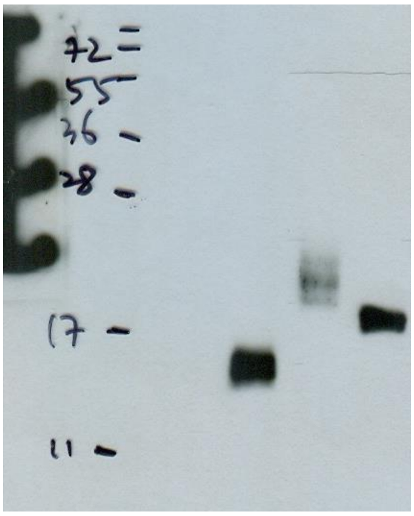

3. In Figure 4D

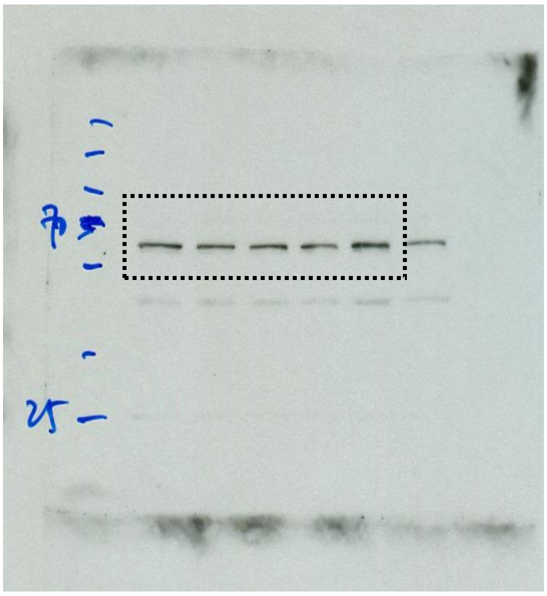

Akt

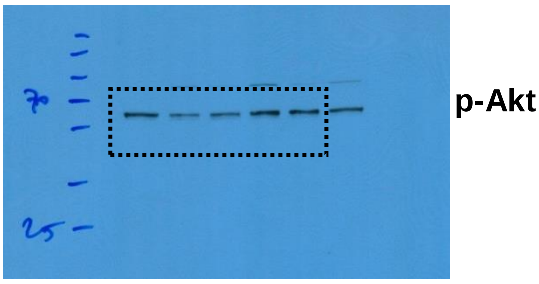

p-Akt

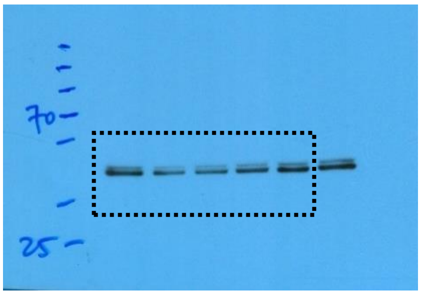

p-ERK

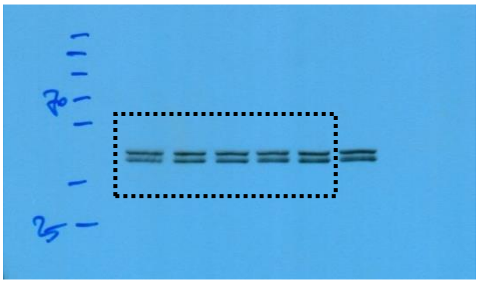

ERK

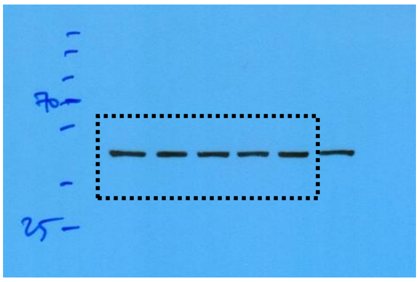

β-actin

4. In Figure 5A

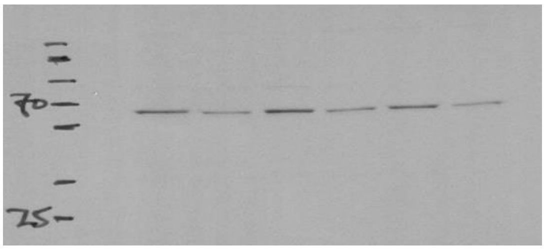

p-Akt

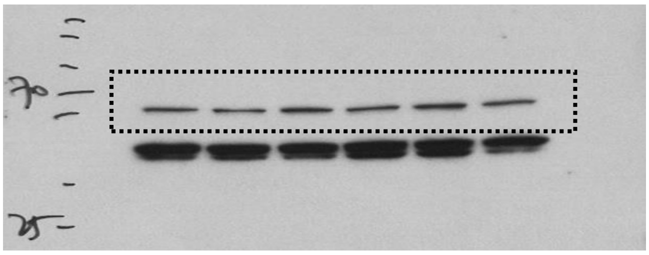

Akt

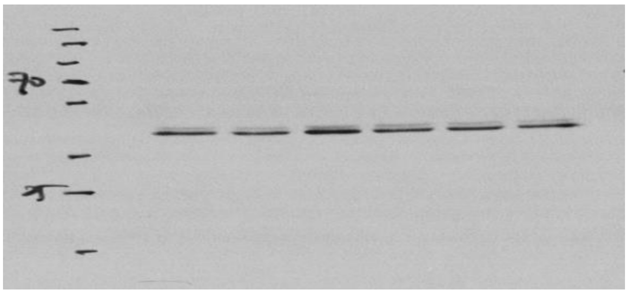

p-ERK

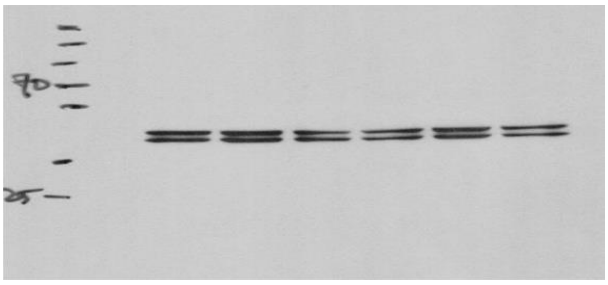

ERK

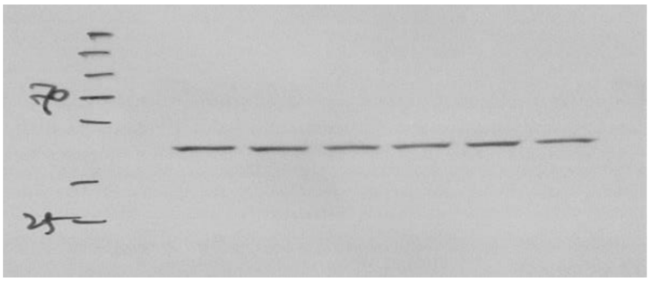

β-actin
